# In Vitro Characteristics of Canine Primary Tracheal Epithelial Cells Maintained at an Air–Liquid Interface Compared to In Vivo Morphology

**DOI:** 10.3390/ijms24054987

**Published:** 2023-03-05

**Authors:** Sandra Runft, Iris Färber, Johannes Krüger, Kerstin Schöne, Annika Lehmbecker, Wolfgang Baumgärtner

**Affiliations:** Department of Pathology, University of Veterinary Medicine, Foundation, 30559 Hannover, Germany

**Keywords:** primary cell cultures, respiratory tract, air–liquid interface, canines, tight junctions

## Abstract

Culturing respiratory epithelial cells at an air–liquid interface (ALI) represents an established method for studies on infection or toxicology by the generation of an in vivo-like respiratory tract epithelial cellular layer. Although primary respiratory cells from a variety of animals have been cultured, an in-depth characterization of canine tracheal ALI cultures is lacking despite the fact that canines are a highly relevant animal species susceptible to various respiratory agents, including zoonotic pathogens such as severe acute respiratory coronavirus 2 (SARS-CoV-2). In this study, canine primary tracheal epithelial cells were cultured under ALI conditions for four weeks, and their development was characterized during the entire culture period. Light and electron microscopy were performed to evaluate cell morphology in correlation with the immunohistological expression profile. The formation of tight junctions was confirmed using transepithelial electrical resistance (TEER) measurements and immunofluorescence staining for the junctional protein ZO-1. After 21 days of culture at the ALI, a columnar epithelium containing basal, ciliated and goblet cells was seen, resembling native canine tracheal samples. However, cilia formation, goblet cell distribution and epithelial thickness differed significantly from the native tissue. Despite this limitation, tracheal ALI cultures could be used to investigate the pathomorphological interactions of canine respiratory diseases and zoonotic agents.

## 1. Introduction

The respiratory tract is in close and continuous contact with the outer environment. Through the gaseous exchange, myriads of airborne pathogens directly get in touch with the respiratory epithelium [1]. To prevent these organisms from causing insults, the respiratory epithelium provides a physicochemical barrier and builds, together with the innate immune system, the first line of defense [2]. In order to study host-pathogen interactions in the respiratory tract, it is essential to gain comprehensive knowledge about the characteristic cellular structure and the complexity of the respiratory tract epithelium. The latter consists of a diverse range of cell types, including basal cells, ciliated cells, brush cells, goblet cells, club cells, neuroendocrine cells, ionocytes and Hillock cells, which vary in concentration throughout the respiratory tract and form a pseudostratified, columnar epithelium [3,4,5]. Tight junctions are intercellular, junctional complexes expressed on the apical side of epithelial cells to maintain the integrity of the airway epithelium, which is necessary for an impermeable barrier function [6]. This physical barrier is enhanced by the mucociliary escalator, which clears the conducting airways by entrapping inhaled particles in mucus derived from secretory cells and propelling the mucus by coordinated ciliary movement anteriorly towards the pharynx [7]. In response to epithelial injury, a subset of basal cells demonstrates the capability of self-renewal and differentiation into ciliated and secretory cells for orderly regeneration and preservation of the protective barrier [8]. 

However, numerous microbes and viruses causing partially severe damage to the host’s respiratory tract have developed different mechanisms to overcome the barrier function in order to transmigrate to deeper tissues. One highly relevant example of this is the severe acute respiratory coronavirus 2 (SARS-CoV-2) as the causative agent of the ongoing coronavirus disease 2019 (COVID-19) pandemic, which demonstrates the far-reaching socio-economic impact of respiratory diseases and outlines the urgent need for in-depth studies concerning the host-pathogen interaction [9]. Such investigations could be used to elucidate the cellular tropism of the pathogen and its transmission-triggered pathology and the possible impact of modulation on pathogen virulence. Special emphasis could be paid to the zoonotic or species-specific impact of the pathogen or both [10]. In this respect, dogs living in close contact with humans represent a species with a high potential to transmit contagious diseases. More importantly, various canine pathogens that affect the respiratory tract, including canine distemper virus [11,12], canine parainfluenza virus [13] and canine herpesvirus [14], as well as *Bordetella bronchiseptica* [15,16], represent candidates that require additional in-depth analysis of their pathogenesis.

In accordance with the 3Rs principle of replace, reduce, refine and with regard to economic and ethical reasons concerning animal experiments; there is an urgent need for reliable and robust in vitro models of the respiratory epithelium that mimic as close as possible the in vivo situation as a useful tool to study pathomorphological processes associated with diseases of the respiratory tract. Primary and immortalized airway epithelial cells cultured as submerged monolayers poorly reflect the multicellular complexity of their in vivo counterparts as they mostly do not develop into secretory and ciliated cells [17,18]. In contrast, airway epithelial cells cultured under air–liquid interface (ALI) conditions with additional growth and differential factors in the culture medium proliferate and differentiate into all main cell types of the respiratory tract, including basal, ciliated and goblet cells and closely mimic the in vivo epithelium [19,20,21,22]. These cultures have been applied in infection studies for a broad variety of species, including humans [23]. Canine airway epithelial cultures maintained under ALI conditions have been successfully established and their application in infection studies with SARS-CoV-2 [24,25], canine distemper virus [26] and *Bordetella bronchiseptica* [27], as well as their use in bioelectric measurements to confirm the sodium-absorbing activities of the epithelium [28], has been preliminarily described. Still, a detailed characterization of canine tracheal epithelial ALI cultures and a correlation to in vivo circumstances have not been published yet. However, this would be an essential prerequisite for a comprehensive evaluation of morphological changes within airway ALI cultures caused by respiratory diseases. The successful application of the described canine air–liquid interface cultures can aid in the reduction of animals experiments for two purposes (i) in vitro instead of in vivo investigations to study host-pathogen interaction in the dog and (ii) to search for potential inter-species transmission is already well-known and new emerging infectious pathogens.

It is the aim of this study to provide a detailed analysis of the morphological characteristics of canine tracheal epithelial ALI cultures maintained for 28 days with special emphasis on squamous proliferation, epithelial thickness, development of tight junctions and mucociliary differentiation in comparison to cells in native tissue. The obtained data enables the identification of an ideal time frame for cultivation and infectious and toxicological investigations of canine ALI cultures in future studies. 

## 2. Results

### 2.1. In Vitro Epithelium Formation of Primary Canine Tracheal Epithelial Cells 

**Figure 1:**
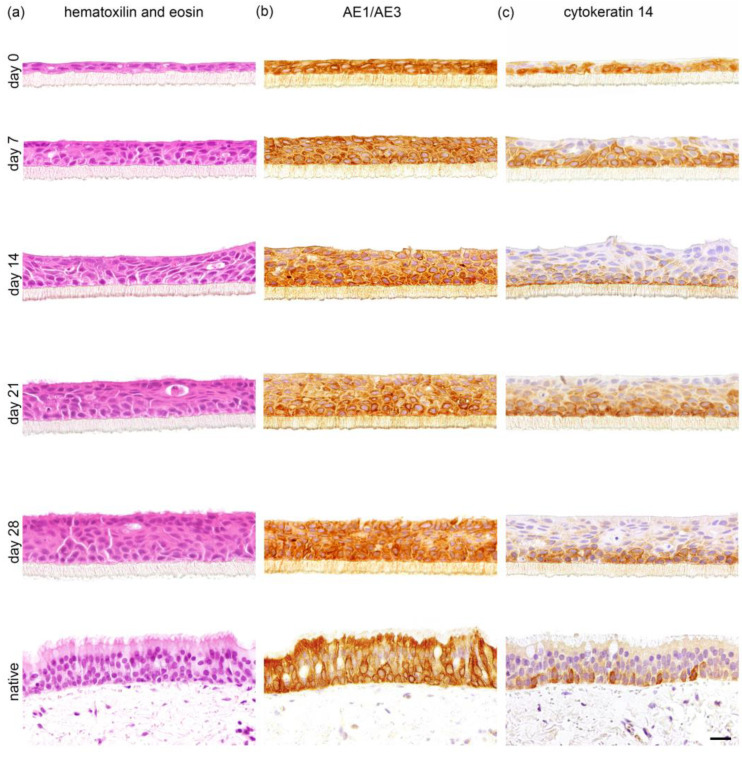
Histological evaluation of tracheal epithelial air–liquid interface (ALI) cultures during a four-week observation period. Primary tracheal epithelial cells were cultured for the indicated number of days (left labeling), then formalin-fixed and paraffin-embedded using standardized protocols. Sections were stained using (**a**) hematoxylin and eosin for evaluation of general cellular morphology, immunohistochemistry with (**b**) pan-cytokeratin (AE1/AE3) to visualize the epithelial cytoskeleton and (**c**) cytokeratin 14 to determine the expression of basal cells. Scale bar represents 20 µm.

**Figure 2:**
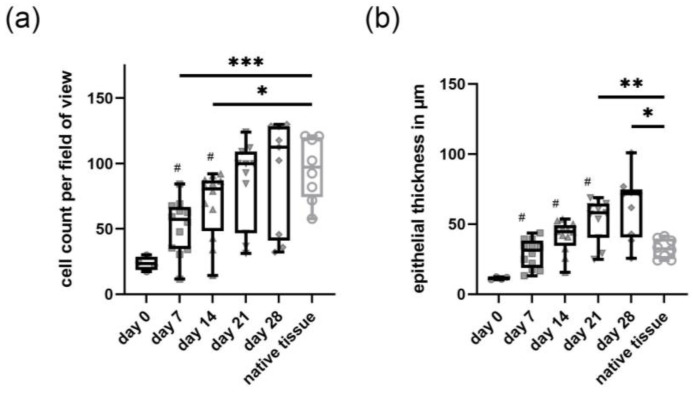
Statistical analysis of cell count and epithelial thickness of primary tracheal epithelial cells maintained at an air–liquid interface (ALI) at different time-points in comparison to native tissue: Quantitative analysis was performed using hematoxylin and eosin stained sections. (**a**) Epithelial cells were counted, and (**b**) epithelial thickness was measured in five fields at a 200× magnification per section evenly distributed across the strand. Two inserts were analyzed per time-point, and the data represent the mean ± standard deviation using tissues derived from different animals (see also Table S1). Significance was analyzed with the one-way-ANOVA and Tukey multiple comparison test. Increasing trends over time and in comparison to the native tissue for both (**a**) cell count and (**b**) epithelial thickness were demonstrated to be significant (* *p* < 0.05, ** *p* < 0.01, *** *p* < 0.001) with “#” meaning significant increase compared to the time-point before.

During the submerged culture conditions (day 6 to day 0), the primary canine tracheal epithelial cells formed a squamous mono- or double layer, which developed during the following 28 days under ALI conditions to a well-differentiated, multi-layered, pseudostratified epithelium. In the hematoxylin and eosin (H&E) staining, the epithelium appeared to be thicker than the native tracheal epithelium, with multifocal areas of cytoplasmic vacuolation and a generalized disarrangement of cells (Figure 1a). Histologically, epithelial cells showed a polygonal form with scant eosinophilic cytoplasm and mostly indistinct cell borders. These cells contained one oval, mostly central to the paracentral nucleus, with finely stippled chromatin. Both the in vitro as well as the native tracheal epithelium showed a distinct, positive immunohistochemical staining with the pan-cytokeratin antibody (clone AE1/AE3) (Figure 1b), which constitutes a marker for epithelial cells. From day 7 up to day 28 under ALI conditions, a distinct single-cell layer composed of small-sized, roundish-shaped cells with round to oval, medium-sized nuclei and small amounts of homogeneous cytoplasm was presently attached to the insert membrane, which was identified as basal cells. The presence of basal cells in the cultures was additionally confirmed by immunohistological staining for cytokeratin 14 (CK14, Figure 1c). Similar findings regarding epithelial and basal cells were observed in the native tracheal epithelium (Figure 1b,c, lower row). Comparing the total cell count in the H&E staining, there was a significant, time-dependent increase in the number of cells from day 0 to day 21 post-initiation of ALI. There was no significant difference between the number of cells from day 21 and day 28 compared to native tracheal tissue indicating that primary canine tracheal epithelial cells at ALI reached a relatively stable plateau after 21 days with a median of 100 cells per high power field compared to a median of 97 cells per high power field in native tracheal tissue (Figure 2a). Similarly, the epithelium grown at ALI gradually increased in thickness from a mean of 11.43 µm at day 0 to a mean of 62.52 µm (ranging from 25.72 µm up to 100.96 µm) after 28 days measured in the H&E staining. In comparison, the native tissue displayed a mean thickness of 32.59 µm with a minimum of 24.20 µm and a maximum of 41.49 µm (Figure 2b).

### 2.2. Development of Barrier Function in Primary Canine Tracheal Epithelial Cells

**Figure 3:**
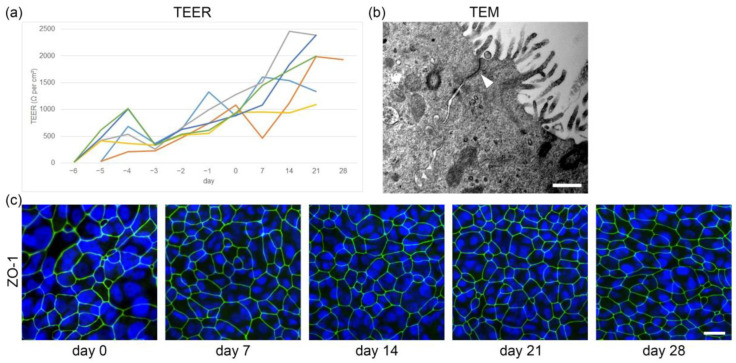
Assessment of tight junctions expressed in primary tracheal epithelial cells under air–liquid interface (ALI) conditions. (**a**) The formation of tight junctions during primary tracheal epithelial cell proliferation and differentiation was confirmed using transepithelial electrical resistance (TEER) measurements in ALI cultures derived from six individual dogs, ranging from day 6 to day 28 after initiation of ALI conditions. Each color of displayed lines represents values of an individual animal. (**b**) Tight junctions (arrowhead) along the epithelial cell borders were observed using transmission electron microscopy (TEM). Scale bar represents 500 nm. (**c**) ALI cultures were grown for the indicated number of days at ALI and fixed on the membranes. Immunofluorescence staining was performed with an anti-zonula occludens-1 (ZO-1) antibody labeling tight junctions in green and nuclear counterstain in blue. Representative images are shown of ALI cultures at days 0, 7, 14, 21 and 28. Scale bar represents 20 µM.

During the submerged growth phase, the epithelial cells form a contiguous squamous epithelium with the development of tight junctions and changes in barrier function. The increasing progression of this process was evaluated using transepithelial electrical resistance (TEER) measurements (Figure 3a). On the first day after seeding on inserts, low levels of TEER values under ~50 Ω per cm^2^ were detected. After 5 to 6 days of submerged culture (day 0 after initiation of ALI conditions), TEER increased rapidly with a mean of 1.000 Ω per cm^2^ (ranging from 878 to 1282 Ω per cm^2^), indicating the formation of an intact cellular barrier. This finding coincided with the observed lack of leakage of culture medium from the basal to the apical compartment when ALI conditions were generated. After the establishment of the ALI, TEER values gradually rose up to a maximum of 2459 Ω per cm^2^ at day 14 in one animal and stabilized at a mean of 1657 Ω per cm^2^ between day 14 and 28. In one animal, TEER values drastically declined at day 7 post initiation of ALI conditions (463 Ω per cm^2^), but the concerning culture macroscopically did not show a decrease in barrier function since no leakage from the basal compartment through the cell layer was observed. Accordingly, TEER values in the ALI culture of this animal stabilized at ~1950 Ω per cm^2^ between day 21 and 28. The presence of tight junctions was further confirmed by transmission electron microscopy, where junctional complexes were identified from day 7 post initiation of ALI conditions onwards (Figure 3b). 

Additionally, epithelial integrity was assessed with immunofluorescence staining using the junctional protein zonula occludens-1 (ZO-1) as a marker for tight junctions (Figure 3c). The latter was present in primary canine tracheal epithelial cells at ALI during all investigated time points. Corresponding with the expanding number of cells present during the culture period of up to 28 days, the development of tight junctions increased and the morphology of ZO-1 positive cells changed from a large, round to oval to a smaller, more polygonal pattern. 

### 2.3. In Vitro Ciliogenesis in Primary Canine Tracheal Epithelial Cells

**Figure 4:**
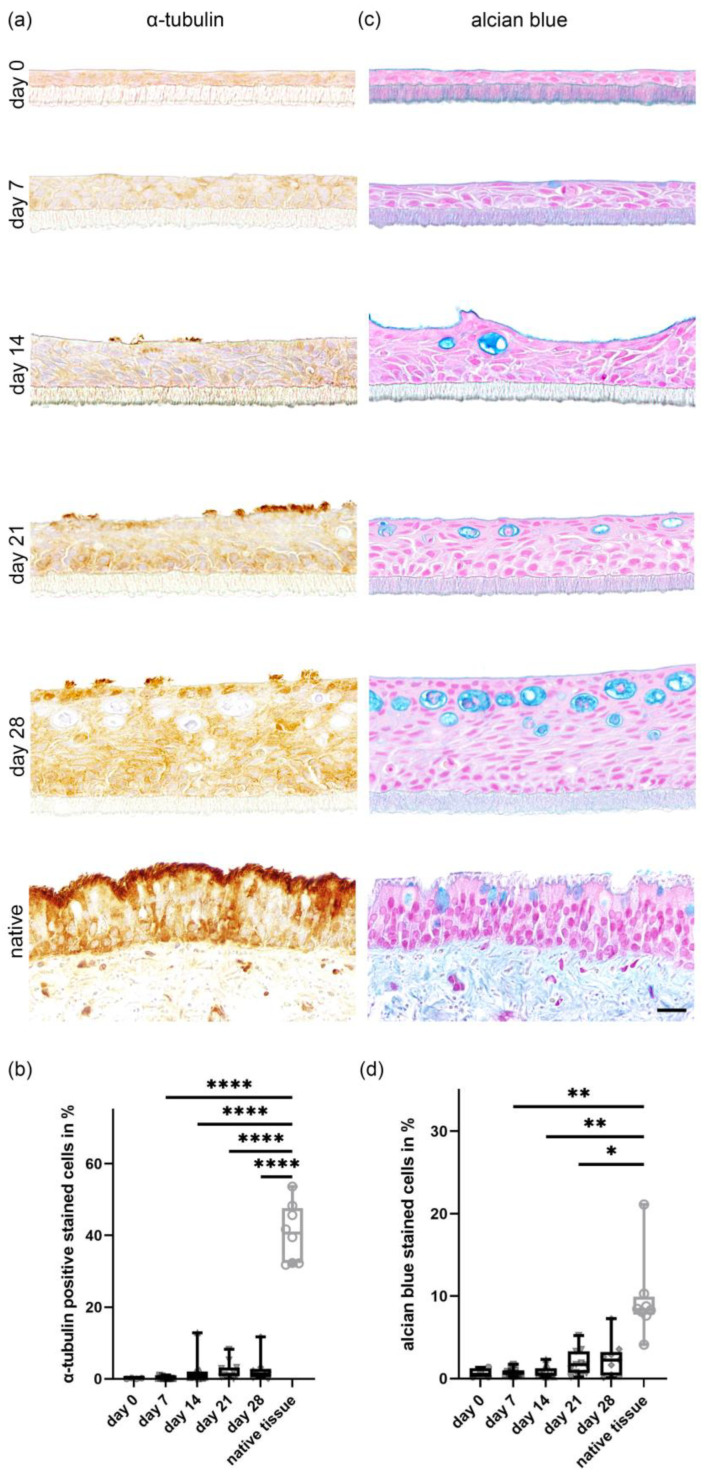
Characterization of mucociliary differentiation of respiratory epithelial cells cultured under air–liquid interface (ALI) conditions using histochemistry and immunohistochemistry. (**a**) Immunohistochemical staining for α-tubulin was performed in order to quantify the number of ciliated cells present in the cultures. Fully-formed cilia were present at day 14, with an increasing number during the following time points. The highest number of ciliated cells was seen in cultures at day 28 post-initiation of ALI conditions. (**b**) Ciliated cells were quantified by counting α-tubulin positive cells in five fields of view per section at 200× magnification. (**c**) Alcian blue staining was applied to visualize mucus-producing goblet cells, with a maximal amount present at day 28 post-initiation of ALI conditions. Goblet cells seemed to be located more often in the middle and upper layer of the epithelium grown at ALI starting from day 14 until day 28. (**d**) Quantitative analysis of mucus-producing goblet cells was performed by counting the number of alcian blue positive stained cells in five fields of view at 200× magnification. For all of the above quantifications, two inserts with two sections each were analyzed per time point, and the data represent the mean ± standard deviation from tissue derived from different animals (see also Table S1). * *p* < 0.05, ** *p* < 0.01, **** *p* < 0.0001 by one-way-ANOVA and Tukey multiple comparison test. Scale bar represents 20 µM.

**Figure 5:**
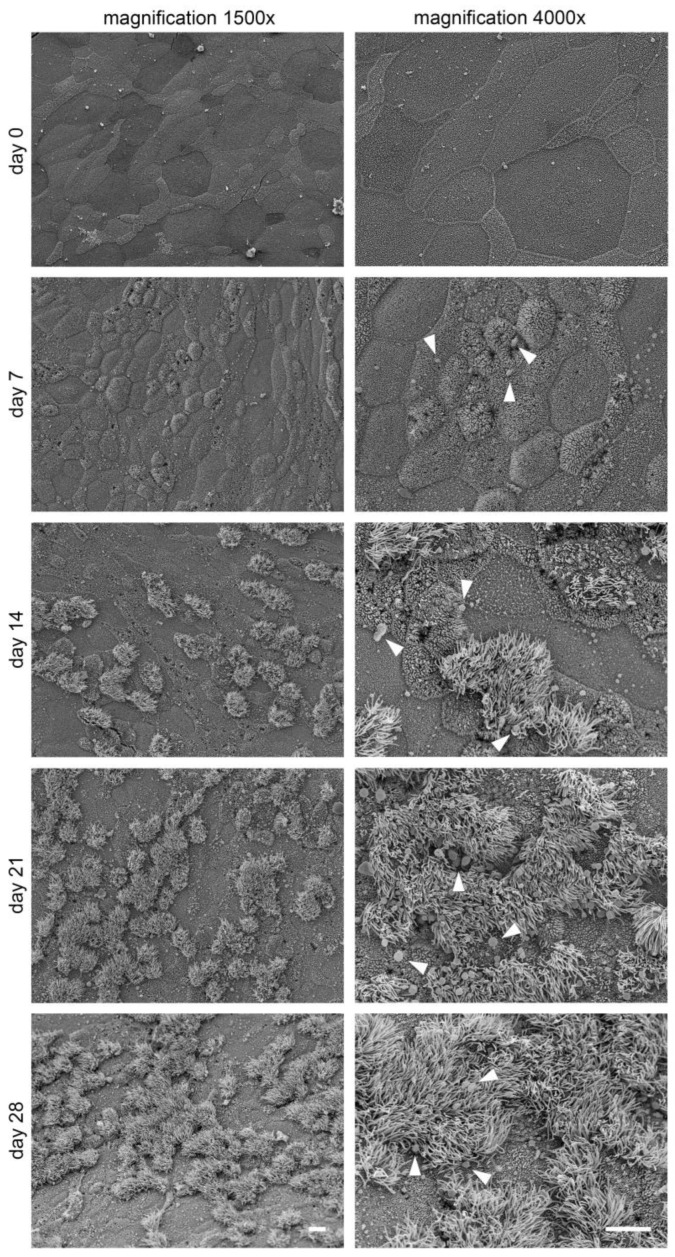
Scanning electron microscopic (SEM) assessment of cilia differentiation in tracheal epithelial cells under air–liquid interface (ALI) conditions over time. Representative pictures are shown of ALI cultures at days 0, 7, 14, 21, and 28. At day 0, undifferentiated tracheal epithelial cells were present with a flat, apical surface. Increasing numbers of ciliated cells were seen over the culture period starting from day 7 up to day 28. Cilia were arranged in individual bundles on the surface of epithelial cells, often along with numerous microvilli that were also frequently present. Additionally, SEM revealed the presence of mucus globules (arrowheads) starting from day 7. Pictures in the left column are taken at 1500× and in the right column at 4000× magnification. Scale bars represent 10 µM.

**Figure 6:**
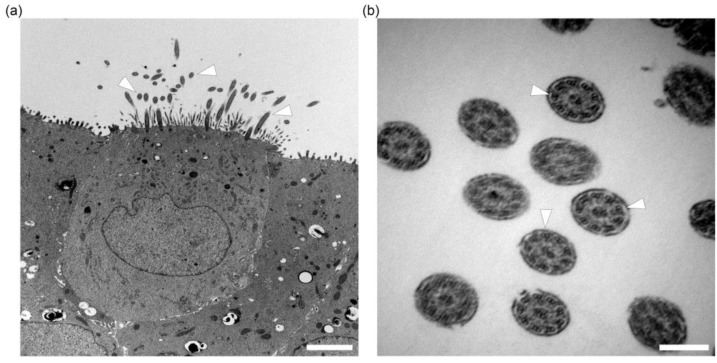
Ultra-structural analysis of cilia formation in differentiated tracheal epithelial cells under air–liquid interface (ALI) conditions. (**a**) Representative image of an ALI culture at day 21 with fully developed cilia (arrowheads). Cilia show longitudinal and unidirectional growth varying in length and diameter with basal bodies. Scale bar represents 2500 nm. (**b**) Higher magnification (scale bar represents 250 nm) of cilia in cross sections with 9 + 2 axoneme arrangement (arrowheads) within cilia membrane.

The development of ciliated cells on the apical surface in correlation to the time in culture was investigated by light microscopy using H&E stained sections and immunohistochemistry. Ciliated cells were visualized using a marker for α-tubulin, a protein that can be found in the cytoskeleton of cilia (Figure 4a). On day 0, post-establishment of the ALI conditions, only a few ciliated cells in cultures in 2 out of 12 dogs were observed. After 7 days under ALI conditions, cultures of 11 out of 12 dogs expressed microvilli and few short cilia with a mean of 4.54 ciliated cells. At day 14, 21 and 28 of post-generation of ALI conditions, fully-formed cilia were present in an increasing number, with a maximum value of 11.67% of ciliated cells in one animal at day 28. ALI cultures from one dog did not show any cilia until day 28. A mean value of 2.54% (range from 0.06% up to 11.67%) of ciliated cells was seen after 28 days compared to the total number of cells. In comparison, the native tracheal epithelium displayed a mean of 40.64% of ciliated cells (Figure 4b). Increasing numbers of ciliated cells were seen during the entire culture period using scanning electron microscopy. Cilia were arranged in individual bundles on the surface of epithelial cells, often displaying a unidirectional growth. Several developmental stages of cilia varying in length and diameter could be identified, along with numerous microvilli that were also frequently present (Figure 5). In transmission electron microscopy, fully developed cilia with basal bodies in longitudinal and 9 + 2 axoneme structures in cross sections were seen at day 14 in culture under ALI conditions and at all later evaluated time points (Figure 6). 

### 2.4. In Vitro Differentiation of Goblet Cells Derived from Primary Tracheal Epithelial Cells

The differentiation of goblet cells with mucus production was assessed over the 28-day-period of ALI growth using H&E, alcian blue-, periodic acid-Schiff (PAS)- and mucicarmine-stain as well as immunohistological staining using the protein mucin 5AC. Since the alcian blue staining showed the most clearly evaluable results, only data from this staining method were further analyzed (Figure 4c). In the native tracheal tissue, a mean of 9.6% of the total counted cells were goblet cells (Figure 4d). Starting from day 0 after initiation of ALI conditions, primary canine epithelial cells displayed a slight, progressive increase in goblet cell formation and mucus production. Starting from a mean value of 0.65% (ranging from 0.18% to 1.38%) of alcian blue positive cells on day 0, values increased to a maximum of 7.26% and a minimum of 0.12% (2.35% of alcian blue stained cell in the mean) on day 28. Well-differentiated goblet cells with their typical round to longitudinal–oval morphological appearance were observed at the apical part of the epithelium on day 7. However, from day 14 until day 28, goblet cells seemed to be located more often in the middle and upper layer of the epithelium grown at ALI and contained horizontal-oval oriented, variably sized vacuoles, possibly indicating cellular degeneration. Secreted mucus was observed using scanning electron microscopy in cultures starting from day 7 post-ALI. The secreted mucus was present in the form of globules that were frequently associated with cilia (Figure 5).

## 3. Discussion

The respiratory epithelium consists of a wide variety of individual cell types, which vary in number and presence depending on the section of the respiratory tract. Major cell types include basal, suprabasal, secretory (club and goblet), ciliated and mucous-ciliated cells. There are also more infrequent cell types present in the airway epithelium, such as ionocytes, pulmonary neuroendocrine, tuft/brush and “Hillock” cells [5,29,30]. In the present study, the aim was to characterize the development of canine tracheal epithelial cells cultured under ALI conditions for 28 days in order to optimize the cultivation of these cells for future application in in vitro experiments. To confirm the epithelial origin of the cells and their differentiation status, a pan-cytokeratin (AE1/AE3) marker was used in the present study. To further characterize the development and differentiation of the epithelial cells, future studies should include additional markers such as CK5 [31], CK6 [31], CK7 [32] and CK18 [31]. This would allow a more precise scoring and staging system of the epithelial cells after seeding for up to 28 days in ALI culture. The absence of mesenchymal cells, such as fibroblasts in the respiratory epithelium in ALI cultures, was verified in two studies using anti-vimentin-specific antibodies [31,33]. Still, other mesenchymal cells might be present, and this should be investigated in future studies with special emphasis on MHC class II expression [34]. The present investigation reveals that culture at an ALI for a minimum of 21 days enables the development of a fully differentiated respiratory epithelium in canines. Importantly, ALI cell cultures showed great similarities and, at the same time, morphological differences compared to native tracheal tissue, regarding, for example, the number of individual cell types such as ciliated cells, distribution of goblet cells and epithelial thickness, which have to be taken into consideration as a limiting factor. 

Basal cells, characterized by positive staining with anti-cytokeratin 14 [35], were frequently found in ALI cultures covering the semipermeable membrane. Cozens et al. (2018) also described the formation of a single layer of basal cells between the epithelium and the insert membrane in bovine bronchial ALI cultures, visualized with an anti-p63 antibody [19], whether this also applies to canine ALI cultures needs to be investigated in further studies. Basal cells are progenitor cells for the airway epithelium, which are located in vivo on the basal lamina of the trachea and pulmonary airways, with species-specific differences in distribution patterns [8,36,37,38,39]. As progenitors, tracheal basal cells are able to differentiate into several cell types, including ciliated and secretory cells [38,39], making their presence essential for the cultivation of a fully developed respiratory epithelium model. However, they also seem to have additional functions regarding the epithelium’s response to infections with viral agents. In another study, an increased proliferation activity of basal cells was reported in the human trachea and large airways as determined by increased expression of Ki67 after SARS-CoV-2 infection. This was interpreted as an attempted regeneration of the epithelium by the basal cells prompted by epithelial damage due to the infection [38]. Additionally, expression of antimicrobial RNase 7 by basal cells was observed after experimental infection of human bronchial epithelial cells with the respiratory pathogen nontypeable *Haemophilus influenzae* [40]. Therefore, the behavior of CK14-positive basal cells in canine ALI cultures following experimental infection with viral and bacterial agents could reveal previously unknown mechanisms of the host’s immune response and should be considered in further investigations for in-depth pathogenetic studies.

The presence of ciliated cells is essential, seeing as ciliary beating together with the mucus layer produced by secretory cells serves as a primary defense mechanism against pathogens through the mucociliary clearance in vivo [19,41]. A multitude of pathogens, such as bacteria [27,42,43,44] and viruses [45,46,47], seems to affect ciliated cells specifically. Ciliated cells were detected via histology and immunohistochemistry using anti-α-tubulin and electron microscopic methods starting from day 7, and their number increased during the following weeks, although the total percentage of the ciliated epithelium at day 28 was generally less than the percentage in native tracheal tissue. Similarly, it was shown by using an anti-β-tubulin specific antibody that bovine primary bronchial epithelial cultures maintained under ALI conditions developed ciliation from day 6 until the end of the culture period, with increasing amounts [19]. Compared to tracheal tissue, however, there were fewer and not as frequently distributed ciliated cells [19]. Another study using human-derived bronchial epithelial cells cultured under ALI conditions and native human tracheal tissue also described a discrepancy, including a lower density of ciliated cells in vitro compared to the in vivo characteristics [48]. Accordingly, a lack of ciliation on a respiratory epithelial ALI culture could reduce the validity of an in vitro infection study performed. Previous studies have shown that the concentrations of various supplements in ALI culture media can significantly influence the differentiation of the cultures, including the presence of ciliated cells [49,50,51,52]. Moreover, another study has shown that inhibition of the Notch signaling pathway with *N*-[*N*-(3,5-difluorophenacetyl)-l-alanyl]-*S*-phenylglycine *t*-butyl Ester (DAPT) can enhance the percentage of ciliated cells in mouse and human airway epithelia [53]. Therefore, further studies are needed to optimize culture media according to each specific species in order to achieve increased ciliation in tracheal epithelial ALI cultures. The used polycarbonate membranes of the porous support chambers were only translucent and not transparent or allowed; therefore, no evaluation of the ciliary beating frequency. However, such a functional analysis is warranted and should be considered in future studies as described for precision cut lung slice analysis [23].

The presence of goblet cells in the ALI cultures of this study was confirmed using alcian blue staining, with few positive cells until day 21 under ALI conditions and increasing amounts thereafter. However, alcian blue-stained goblet cells of the present study differed in shape and localization within the epithelium in comparison to their in vivo counterparts. This discrepancy between in vitro and in vivo goblet cells has not been described so far and is most likely due to different culture conditions. Goblet cells can be defined as specialized epithelial cells responsible for the production of mucus, which is then secreted into the lumen of large and small airways [54,55]. This mucus is essential for the protection against microorganisms and other pathogens in the respiratory tract. Interestingly, the overproduction of mucins due to an amplified immune response and consecutive goblet cell hyperplasia can result in the worsening of the disease progression, for example, seen in chronic bronchitis and obstructive pulmonary disease [56,57,58]. This hypersecretion can be caused by a variety of animate and inanimate noxious agents like cigarette smoke [56,57,58], respiratory viruses [59,60] and bacteria [55,61]. The presence of goblet cells in a respiratory ALI culture is, therefore, highly relevant, especially for in vitro infection studies, to further understand pathomechanisms of toxicological and infectious agents.

The transepithelial electrical resistance (TEER) measurement is a quantitative method to evaluate the integrity of endothelial and epithelial monolayers characterized by the development of tight junctions [62]. In canine ALI cultures of the present study, TEER values measured in Ω showed a continuous increase during the entire culture period. However, single measurements and peak values displayed a high level of variation between individual animals. An initial rapid increase of TEER values was observed during the submerged culture of bovine respiratory epithelial cells, with a decrease in values after the initiation of ALI conditions, which eventually stabilized [19]. A similar finding referring to a mean peak value between 1 and 3 days after ALI initiation was reported in a study using ovine tracheal epithelial cell cultures [63]. Another study performed by culturing porcine respiratory cells mentioned peak values after 6 days under ALI conditions and a mild decrease afterward [64]. Interestingly, little is known about the influence of the species on the development of respiratory epithelial cultures and their respective TEER values. A previous study investigated the differences in TEER of primary brain capillary endothelial cells in several animal species and found that there is a high variation regarding the TEER values of different species [65]. One common feature in all these studies—including the present study described here—was that the tight junctions, visualized by ZO-1 staining, generally remained intact during the whole culture period [19,63,64]. Therefore, the TEER values by themselves might not be fully representative when evaluating tight junction formation. Individual fluctuations in measurements might lead to misinterpretation.

The total cell count of ALI cultures in the present study reached comparable levels to the cell count in native tracheas at day 21, implicating that the proliferation of cultured cells is likely sufficient for further use at this time point. The thickness of the entire cultured epithelium increased throughout the culture period and surpassed the native tracheal tissue at day 14. This expansion of thickness might be due to morphological aberrations between cultured and native epithelial cells: the latter might include an increased number of intracytoplasmic vacuoles, increasing the overall cell size and, therefore, the layer thickness of the epithelium under ALI conditions. Additionally, a mild disarrangement of cultured cells was seen throughout the whole epithelium, leading to variations in the number of cell layers and, therefore, in epithelial thickness. This result was surprising as in other previous studies, the thickness of the cultured epithelium was reduced compared to the epithelium in native tissue [19,48,63]. This might be explained by the different culture protocols that were used, varying concentrations of culture supplements, or might be a species-specific deviation of cultured canine tracheal epithelial cells.

Overall, primary tracheal epithelial cultures derived from dogs were successfully established and cultured for up to 28 days after initiation of ALI conditions, resulting in a well-differentiated, pseudostratified epithelium. Using histological, immunohistochemical and electron microscopic methods, we determined that most characteristic features of the respiratory tracheal epithelium in vivo were maintained in vitro, even though the cellular composition and morphology varied between the native tissue and cell cultures. The latter include lack of cilia formation, the orientation of goblet cells in the late phase of ALI cultures and epithelial thickness. These limitations should be taken into account when planning potential in vitro experiments, considering the objective to be investigated. Despite the fact that the developed canine ALI cultures represent a very useful tool for many investigations, further improvements of the described in vitro model may allow applying primary respiratory cells cultured at an ALI as an even more valuable tool for future investigations to study pathogen cell tropism, spread and replication also in regard to the 3Rs principle.

## 4. Materials and Methods

### 4.1. Collection of Samples 

Samples from a total of twelve dogs were collected for this study in order to obtain formalin-fixed, paraffin-embedded tracheal tissue as well as primary respiratory epithelial cells for further culture. Five canine tracheas were obtained from necropsy cases at the Department for Pathology, University of Veterinary Medicine, Foundation, Hannover, from two male and three female dogs of different breeds, between two months and 13 years of age, free of respiratory diseases. Additional tracheal tissue from four male and three female, 9 months old Beagle dogs was used. Tissue from the Beagle dogs originated from animals that served as healthy control dogs in regulatory animal studies. The regulatory studies were performed in an Association for the Assessment and Accreditation of Laboratory Animal Care (AAALAC)-approved laboratory in accordance with the German Animal Welfare Act and the effective European Council Directive. The studies were approved by the local authorizing agency for animal experiments (Landesuntersuchungsamt Rheinland-Pfalz, Koblenz, Germany) on 18 October 2017, as referenced by the approval number 23 177-07/G 17-3-080. For further details regarding available samples from the individual animals, see Supplementary Material Table S1.

### 4.2. Isolation and Maintenance of Canine Tracheal Epithelial Cell Cultures

The protocol for the isolation of primary tracheal epithelial cells was adapted and slightly modified as previously described [66]. Freshly collected tracheal tissue (<8 h post-mortem) was rinsed with phosphate-buffered saline (PBS-) and transferred to a petri dish under a laminar flow hood. Connective tissue and lymph nodes were removed with a sterile scalpel blade, and the remaining tracheal tissue was rinsed twice with PBS-. Two tracheal rings per donor animal were cut, formalin-fixed and paraffin-embedded. The remaining trachea was transferred to a 50 mL tube filled with washing medium (Supplementary Table S2) and incubated at 4 °C overnight. The next day, the washing medium was replaced by an incubation medium (Supplementary Table S3) for another 24 h. After enzymatic proteolysis, tracheal epithelial cells were harvested with a sterile scalpel blade and transferred to 50 mL tubes filled with Dulbecco’s modified eagle medium (DMEM) with 10% fetal bovine serum (FBS) to neutralize enzyme activity and centrifuged at 250× *g* for 10 min at 4 °C. The supernatant was discharged, and the cell pellet was resuspended in 50 mL PBS-, followed by centrifugation. The cell pellet was then resuspended in BEGM and filtered through a 100 µm porous mesh. Afterward, tracheal epithelial cells were immediately seeded on, depending on pellet size, one or two collagen-I-coated T75 culture flasks and incubated at 37 °C in a humidified 5% CO_2_ incubator. The culture medium was supplemented with additional antibiotics (Supplementary Table S4) and changed 24 h after seeding and then every second to the third day. When the epithelial cells reached 80–90% confluence in the culture flasks, they were washed with PBS and dissociated with 0.05% trypsin-EDTA (7 mL/T75). After 10 min at 37 °C in a humidified 5% CO_2_ incubator, trypsin-EDTA was inhibited with serum-containing culture medium, and the cells were collected in a 50 mL Falcon tube and centrifuged at 250× g for 10 min at 4 °C. The cell pellet was resuspended in PBS-, followed by another centrifugation step. Cells were resuspended in ALI medium (Supplementary Table S5) and counted using a Neubauer counting chamber. Tracheal epithelial cells were seeded at a density of 0.35 × 10^6^ on 24-well plates containing collagen-IV coated, polycarbonate, porous support chambers (inserts) (VWR, Radnor, PA, USA) with 250 µL ALI medium in the apical and 500 µL ALI medium in the basal compartment. The cultures were incubated at 37 °C in a humidified 5% CO_2_ incubator. After 24 h, the medium was removed and cells were washed twice with Hanks’ Balanced Salt Solution (HBSS). Regular medium changes were performed every other day with 200 µL of ALI medium in the apical and 500 µL in the basal compartment. 

### 4.3. Establishment of ALI Cultures

After seeding, canine tracheal epithelial cells were cultured in submerged conditions on inserts, with daily control measurements of the transepithelial resistance (TEER) as previously described [24]. When TEER values indicated confluence of the cellular monolayer, the ALI medium was removed from the apical compartment, with 500 µL of ALI medium remaining only in the basal compartment, establishing ALI conditions. For maintenance, cultures were incubated at 37 °C in a humidified 5% CO_2_ incubator, and the ALI medium was changed every second day, with weekly washing steps with HBSS.

### 4.4. TEER Measurements

For TEER measurements, previous protocols [62] were adapted. The TEER device Millicell^®^ ERS-2 (Merck Millipore, Billerica, MA, USA) was used to verify the integrity of cellular barriers. The chopstick electrode pair was calibrated in ALI medium and placed upright with one electrode in the apical and one in the basal compartment, separated by the layer of cells on the semipermeable membrane. For the measurement procedure, the compartments of two inserts of each dog from a total of six dogs were filled with 500 µL ALI medium in the lower and 200 µL ALI medium in the upper compartment. The resistance across the semipermeable membrane and cell layers (R_total_) was measured in units of Ω daily after seeding until ALI conditions were established. After that, TEER values were measured once a week for up to 28 days post-initiation of ALI conditions. Semipermeable membranes without a cellular monolayer were measured for blank resistance (R_blank_). The tracheal epithelial cell-specific resistance (R_tissue_) was calculated as follows: R_tissue_ (Ω) = R_total_ − R_blank_. TEER values are typically given in units of Ω per cm^2^. Therefore, R_tissue_ was multiplied by the specific area of the semipermeable membranes used in this study, (0.3 cm^2^).

### 4.5. Histochemistry (H&E and Alcian Blue)

For histological examination by light microscopy, native tracheal tissue and inserts with tracheal epithelial cells, which were cultured under ALI conditions, were immersion-fixed at day 0, 7, 14, 21 and 28 in culture in 4% paraformaldehyde (PFA) for at least 24 h and routinely embedded in paraffin wax. For further processing, 2–4 µm thick serial sections were cut from the paraffin wax blocks and routinely stained with hematoxylin and eosin (H&E). 

To visualize mucopolysaccharides in goblet cells, we stained sections with alcian blue as described previously [67]. Briefly, following incubation in 3% acetic acid (Carl Roth GmbH & Co., Karlsruhe, Germany) for two minutes, sections were stained for 2 h in 0.1% alcian blue solution (Chroma Gesellschaft Schmid & Co, Stuttgart, Germany) diluted in 3% acetic acid at pH 2.5. Afterward, the slides were rinsed in tap water, and the nuclei were stained with 0.1% nuclear fast red-aluminum sulfate solution (Merck KGaA, Darmstadt, Germany).

Additionally, intracellular mucus was stained with periodic acid-Schiff (PAS) reaction after Mc Manus using standard staining protocols [68]. The deparaffinized sections were incubated for 10 min in 1% periodic acid (Carl Roth GmbH & Co., Karlsruhe, Germany). Afterward, sections were stained in Schiff reagent (Carl Roth GmbH & Co., Karlsruhe, Germany) for 20 min and rinsed with hot tap water. Following this, sections were immersed in hematoxylin (Carl Roth GmbH & Co., Karlsruhe, Germany) for 5 min, rinsed with tap water and rehydrated. 

For staining procedures with mucicarmine, the sections were immersed in hematoxylin (Carl Roth GmbH & Co., Karlsruhe, Germany) for 5 min. For the mucicarmine stain, a stock solution was prepared with 1 g carmine (Merck KGaA, Darmstadt, Germany), 0.5 g aluminum chloride (Merck KGaA, Darmstadt, Germany), 2 mL distilled water and 100 mL 50% ethanol. The stock solution was diluted 1:10 with 70% ethanol as a working solution in which the sections were stained for 10 min.

### 4.6. Immunohistochemistry and Immunofluorescence

Immunohistochemistry of PFA-fixed, paraffin-embedded tracheal tissue and tracheal epithelial ALI cultures were performed to characterize the epithelial architecture further using the avidin–biotin–peroxidase complex (ABC; Vector Laboratories, Burlingame, CA, USA) method as previously described [24]. The cytoskeleton of the epithelial tissue was investigated through the antibody cocktail AE1/AE3 (Dako, Berlin, Germany, M3515, 1:500) and the expression of basal cells using anti-cytokeratin 14 (Thermo Fisher Scientific, Waltham, MA, USA, RB-9020-P, 1:500). For the detection of mucus-secreting goblet cells anti-Muc5AC (Acris, AM50143P-T, 1:2000) and for ciliated cells, anti-α-tubulin (Sigma Aldrich, St. Louis, MO, USA, T6793, 1:1000) were used. Tissue sections were viewed with an Olympus BX51 light microscope.

Immunofluorescence staining was performed with anti-zonula occludens-1 (ZO-1) (Invitrogen, Carlsbad, CA, USA, 61-7300, 1:50) to detect tight junctions. Therefore, the inserts were fixed in 4% PFA at room temperature for 20 min. After blocking with 10% goat serum, inserts were placed in 25 µL antibody solution, and 50 µL antibody solution was pipetted in the apical compartment. After an incubation time of 2 h at room temperature, inserts were washed using PBS- with 0.25% TritonX-100 and incubated using the secondary antibody Alexa Fluor 488 goat–anti-rabbit in the same way as with the primary antibody for 2 h in the dark. The nuclei were stained using FluoroshieldTM mounting medium (Sigma-Aldrich, St. Louis, MO, USA) and coverslips. The tissue sections were observed with an Olympus IX70 immunofluorescence microscope, and images were captured with an Orca flash 4.0 camera.

### 4.7. Histological Evaluation of ALI Cultures

At each time point, four sections per dog generated from two ALI cultures were evaluated. For the native tracheal tissue, one cross-section was analyzed completely. Briefly, five randomized fields at a 200× magnification were investigated per section using an Olympus BX51 light microscope and photographed with an Olympus DP72 camera. Based on the captured pictures, the total cell count in the H&E staining, alcian blue stained cells, as well as α-tubulin-positive cells were counted, and the epithelial thickness in the H&E staining was measured using the QuPath-0.3.0 software. For quantification of the number of goblet cells present in each sample, respective slides stained with alcian blue solution were evaluated instead of slides stained with Muc5AC antibody because of the observed improved depiction of individual cells. The percentages of alcian blue-stained goblet cells and the α-tubulin-positive ciliated cells were calculated based on the total cell count in both the ex vivo tracheal tissue and the in vitro primary epithelial cell cultures.

### 4.8. Scanning Electron Microscopy

As previously described [69] and according to the osmium (O)-thiocarbohydrazide (T)-embedding (OTOTO) protocol, ALI cultures were firstly fixed in a solution of 2.5% glutaraldehyde in a 0.1 M sodium cacodylate buffer for at least one day. After several washing steps with 0.1 M sodium cacodylate buffer, cultures were treated alternately with a 1% osmium tetroxide compound diluted 1:1 in cacodylate buffer and a 1% thiocarbohydrazide solution, including washing steps with distilled water in between. Subsequently, cultures were submerged in an ascending alcohol series and ultimately stored in 100% ethanol and isoamyl acetate for two days each, after which critical point drying was used for final dehydration. Following mounting on the sample holder, cultures were sputter coated with 15 nm gold. The samples were examined using a Zeiss EVO 15 scanning electron microscope (Carl Zeiss Microscopy, Jena, Germany) operating with an acceleration voltage of 10 kV.

### 4.9. Transmission Electron Microscopy

Cultures were processed for transmission electron microscopy as previously described [67]. ALI cultures were firstly fixed in a solution of 2.5% glutaraldehyde in a 0.1 M sodium cacodylate buffer for at least one day, after which two washing steps with 0.1 M sodium cacodylate buffer followed, with the treatment of the cultures with a 2% osmium tetroxide compound diluted 1:1 in cacodylate buffer in between. Cultures were then submerged in an ascending alcohol series, and propylene oxide was used as a transition solvent for 100% epoxy resin solution, which was then used together with 2,4,6-Tris-(dimethylaminomethyl)-phenol (DMP-30) for culture-embedding in a heating chamber for six days total. The ALI cultures were analyzed with a Zeiss EM 906 transmission electron microscope (Carl Zeiss Microscopy) at 80 kV.

### 4.10. Statistical Analysis

For statistical analysis, GraphPad Prism software version 9.0.0 was used. Data obtained from the histological evaluation were analyzed for normal distribution with the Shapiro–Wilk test. Significance was analyzed by one-way-ANOVA and Tukey multiple comparison test. Data were given with standard deviation, and differences are indicated via *p*-value *p* < 0.05.

## Data Availability

The data presented in this study are available on request from the corresponding author.

## Supplementary Materials

The following supporting information can be downloaded at: https://www.mdpi.com/article/10.3390/ijms24054987/s1.
