# Supplementary material for: In Vitro Characteristics of Canine Primary Tracheal Epithelial Cells Maintained at an Air–Liquid Interface Compared to In Vivo Morphology
Source: Int J Mol Sci. 2023 Mar 5;24(5):4987. doi: 10.3390/ijms24054987 (PMC10003254; doi:10.3390/ijms24054987)
Supplement: Supplementary file 1 [file ijms-24-04987-s001.zip › ijms-2211882-supplementary.pdf]

Supplementary Materials

In Vitro Characteristics of Canine Primary Tracheal Epithelial Cells Maintained at an Air-Liquid Interface Compared to In Vivo Morphology

Table S1. Overview of dogs used for the collection of native tissue and for the generation of air-liquid interface cultures.

Manuscript animal number	Internal animal number	Sex	Age	Breed	Native tissue	ALI cultures				
						day 0	day 7	day 14	day 21	day 28
dog #1	V957/19	male	9 months	Beagle	X		X	X	X	
dog #2	V958/19	male	9 months	Beagle	X		X	X	X	X
dog #3	V1019/19	female	9 months	Beagle	X	X	X	X	X	X
dog #4	V1020/19	female	9 months	Beagle	X		X	X	X	X
dog #5	V1021/19	female	9 months	Beagle	X		X	X	X	X
dog #6	V1022/19	male	9 months	Beagle	X		X	X	X	X
dog #7	V1023/19	male	9 months	Beagle	X		X	X	X	X
dog #8	S546/20	female	13 years	mixed breed dog	X		X	X	X	X
dog #9	S582/20	female	2 months	Greater Swiss Mountain Dog		X	X	X	X	X
dog #10	S1112/20	female	13 years	Labrador Retriever			X	X	X	X
dog #11	S773/21	male	4 months	Cane Corso Italiano		X	X	X	X	
dog #12	S1051/21	male	9 years	mixed breed dog		X	X	X		

Table S2. Substances added to Dulbecco's Modified Eagle Medium (DMEM) (Gibco/Fisher Scientific, Waltham, Massachusetts, USA) used as washing medium.

Reagents	Supplier	Final concentration
Penicillin/streptomycin	Sigma-Aldrich, St. Louis, Missouri, USA	1%
Amphotericin B	Sigma-Aldrich, St. Louis, Missouri, USA	2.5 µg/mL

Table S3. Substances added Dulbecco's Modified Eagle Medium (DMEM) (Gibco/Fisher Scientific, Waltham, Massachusetts, USA) used as incubation medium.

Reagents	Supplier	Final concentration
Penicillin/streptomycin	Sigma-Aldrich, St. Louis, Missouri, USA	1%
Amphotericin B	Sigma-Aldrich, St. Louis, Missouri, USA	2.5 µg/mL
Protease	Sigma-Aldrich, St. Louis, Missouri, USA	1 mg/mL
DNase	Roche, Basel, Switzerland	0.01mg/mL

Table S4. Substances added to bronchial epithelial cell growth basal medium (BEBM) (Lonza, Basel, Switzerland) used as bronchial epithelial cell growth medium (BEGM).

Reagents	Supplier	Final concentration
Fetal calf serum	Capricorn Scientifics, Ebsdorfergrund, Germany	10%
Penicillin/streptomycin	Sigma-Aldrich, St. Louis, Missouri, USA	1%
Bovine pituitary extract	Sigma-Aldrich, St. Louis, Missouri, USA	14µg/mL
Bovine serum albumin	Sigma-Aldrich, St. Louis, Missouri, USA	500µg/mL
Insulin	Sigma-Aldrich, St. Louis, Missouri, USA	5µg/mL
Transferrin	Sigma-Aldrich, St. Louis, Missouri, USA	20µg/mL
Hydrocortisone	Sigma-Aldrich, St. Louis, Missouri, USA	72ng/mL
Triiodothyronine	Sigma-Aldrich, St. Louis, Missouri, USA	67ng/mL
Epinephrine	Sigma-Aldrich, St. Louis, Missouri, USA	0.6µg/mL
Epidermal growth factor	BD Biosciences, Franklin Lakes, New Jersey, USA	50ng/mL
Retinoic acid	Sigma-Aldrich, St. Louis, Missouri, USA	0.001nM/mL
Phosphorylethanolamine	Sigma-Aldrich, St. Louis, Missouri, USA	70µg/mL
Ethanolamine	Sigma-Aldrich, St. Louis, Missouri, USA	30nL/mL

Table S5. Culture medium additives for maintaining cells under air-liquid interface conditions based on 50% BEBM (Lonza, Basel, Switzerland) and 50% DMEM (Gibco/Fisher Scientific, Waltham, Massachusetts, USA).

Reagents	Supplier	Final concentration
Fetal calf serum	Capricorn Scientifics, Ebsdorfergrund, Germany	10%
Penicillin/streptomycin	Sigma-Aldrich, St. Louis, Missouri, USA	1%
Bovine pituitary extract	Sigma-Aldrich, St. Louis, Missouri, USA	14µg/mL
Bovine serum albumin	Sigma-Aldrich, St. Louis, Missouri, USA	500µg/mL
Insulin	Sigma-Aldrich, St. Louis, Missouri, USA	5µg/mL
Transferrin	Sigma-Aldrich, St. Louis, Missouri, USA	20µg/mL
Hydrocortisone	Sigma-Aldrich, St. Louis, Missouri, USA	72ng/mL
Triiodothyronine	Sigma-Aldrich, St. Louis, Missouri, USA	67ng/mL
Epinephrine	Sigma-Aldrich, St. Louis, Missouri, USA	0.6µg/mL
Epidermal growth factor	BD Biosciences, Franklin Lakes, New Jersey, USA	1ng/mL
Retinoic acid	Sigma-Aldrich, St. Louis, Missouri, USA	0.001nM/mL
Phosphorylethanolamine	Sigma-Aldrich, St. Louis, Missouri, USA	70µg/mL
Ethanolamine	Sigma-Aldrich, St. Louis, Missouri, USA	30nL/mL
